# Successful Management of Presumed Reactivation of *Neospora caninum* Following Immunosuppression for Immune Thrombocytopenia in an Adult Doberman

**DOI:** 10.1002/vms3.70425

**Published:** 2025-05-28

**Authors:** Perrine Henry, Paul Rees, Tyler Morrison, Glynn Woods

**Affiliations:** ^1^ Internal Medicine Department The University of Edinburgh Hospital for Small Animals (HfSA), Royal (Dick) School of Veterinary Studies (R(D)SVS) Midlothian UK

**Keywords:** dogs, parasites, *Neospora caninum*

## Abstract

A 5‐year old, vaccinated, untravelled and neutered female Doberman with occasional exposure to raw food presented for investigation of severe thrombocytopenia and anaemia. Extensive investigations led to a diagnosis of non‐associative immune thrombocytopenia, with secondary bleeds resulting in anaemia. Management with repeated packed red blood cell transfusions, vincristine (0.02 mg/kg single intravenous injection), prednisolone (50 mg/m^2^/day) and mycophenolate (10 mg/kg twice daily) allowed normalisation of haematology parameters. Scheduled reassessment 10 days after discharge revealed lethargy, ataxia, wide‐based stance and marked generalised muscle wastage. Platelet count remained normal, but biochemistry was supportive of muscle damage. *Neospora caninum* antibodies were elevated (indirect fluorescence antibodies > 1600). Rapid tapering of prednisolone alongside clindamycin (20 mg/kg twice daily) allowed a complete recovery. Mycophenolate was slowly tapered and discontinued after 12 weeks, as was antibiotic therapy. This case describes the successful management of a dog with presumed neuromuscular neosporosis, suspected secondary to reactivation of the parasite from immunosuppression, and absence of long‐term complications.

## Graphical abstract

Positive serology titres for *Neospora caninum* in the absence of appropriate clinical signs is not diagnostic for protozoal disease. Acute deterioration while immunosuppressed should raise concerns over reactivation of encysted protozoa, particularly in dogs with a history of raw feeding. Successful management of *N. caninum* appears possible but requires early identification. Clindamycin may be sufficient to treat neuromuscular *N. caninum*, particularly in breeds with known intolerance or at high risk of developing keratoconjunctivitis sicca, hypothyroidism or drug idiosyncrasies with trimethoprim sulfamethoxazole.

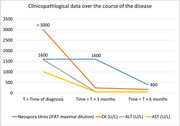

## 1 Introduction

*Neospora caninum* is an intracellular protozoan responsible for neosporosis, an infectious disease resulting in neuromuscular disorders in dogs worldwide, and carries significant one health concerns as a cause of neonatal mortality and abortion in cattle (Donahoe et al. 2015). Seroprevalences are reported between 0% and 76% across the world depending on the population of dogs tested (urban fed a commercial diet vs. farm dogs or raw fed) (Dubey et al. 2007). Diagnosis of neosporosis relies on identification of compatible clinical signs and parasite detection, either by direct methods (visualisation of tachyzoites in tissues, fluid or cytopathological samples) or indirect methods (serology) (Silva and Machado 2016; Alf et al. 2024).

Description of the clinical course of neosporosis is limited to case reports and small case series (Barker et al. 2021; Fisher et al. 2024). Several presentations exist depending on the mode of transmission and age of infection. Vertical transmission, whether prenatal (trans‐uterine) or post‐natal (trans‐mammary) in puppies, presents either as ascending limb paralysis followed by gradual muscular atrophy and often irreversible rigid contraction of the affected muscles or, in some cases, as asymptomatic carriage contributing to the perpetuation of infected bloodlines (Barber and Trees 1998; Sykes et al. 2021; Morganti et al. 2024). Adult dogs are typically infected by ingestion of tissue cysts (raw meat, predation) but usually asymptomatic (Silva and Machado 2016). When clinical, adult‐onset neosporosis presents with signs reflecting the involvement of the central nervous system, the peripheral nervous systems or both (flaccid paralysis, paresis, ataxia, and encephalomyelitis) and occasionally myositis or rhabdomyolysis (Silva and Machado 2016; Alf et al. 2024; Fisher et al. 2024; Sykes et al. 2021). Other forms of the disease (systemic, hepatic and cutaneous) are uncommon and have only been reported in association with immunosuppression (La Perle et al. 2001 May; Hoon‐Hanks et al. 2013; Magaña et al. 2015). Protozoal infections by *Toxoplasma gondii* and *N. caninum* have been described following immunosuppression in cats and dogs (La Perle et al. 2001 May; Hoon‐Hanks et al. 2013; Magaña et al. 2015; Dubey et al. 2020; Curtis et al. 2020; Newton and Manens 2018; Legnani et al. 2016; Galgut et al. 2010; Ordeix et al. 2002; Fry et al. 2009; Serrano and Freeman 2017; Mann et al. 2016; Gupta et al. 2011; Schuyter et al. 2013). Most cases were presumed reactivations of the parasite but primo‐infection with *T. gondii* during immunosuppression is reported to have a very poor outcome. Reactivation of *N. caninum* secondary to immunosuppression has only been described in 14 dogs, of which two were systemic (tachyzoites present in the brain, pancreas, liver, gastrointestinal tract and/or lungs) (Magaña et al. 2015; Curtis et al. 2020), two neuromuscular (Galgut et al. 2010; Serrano and Freeman 2017), three protozoal hepatitis (Hoon‐Hanks et al. 2013; Newton and Manens 2018; Fry et al. 2009) and seven cutaneous forms (La Perle et al. 2001 May; Legnani et al. 2016; Ordeix et al. 2002; Mann et al. 2016; Gupta et al. 2011; Schuyter et al. 2013). Successful management was most reported in cutaneous cases (4/7) (La Perle et al. 2001 May; Legnani et al. 2016; Ordeix et al. 2002; Schuyter et al. 2013), whereas other locations were associated with poor prognosis (death or euthanasia following deterioration). Two more dogs made a recovery, one with protozoal hepatitis and one with neuromuscular neosporosis (Newton and Manens 2018; Serrano and Freeman 2017). Both required rapid withdrawal of all immunosuppression alongside trimethoprim–sulphonamide (TMPS) and pyrimethamine, in combination with clindamycin for the latter case.

To the authors’ knowledge, this is the first report of neuromuscular neosporosis, presumed secondary to protozoal reactivation from immunosuppression, successfully treated while receiving ongoing immunomodulating drugs (mycophenolate) with clindamycin and supportive care (nutrition, physiotherapy).

## 2 Case Presentation

A 5 year‐old, neutered female Doberman was referred for investigations and treatment of thrombocytopenia and anaemia. She was up to date with vaccinations, received regular flea and worming preventatives and was fed a balanced commercial diet with occasional raw meat treats. The dog lived in an urban area of Scotland (United Kingdom), with no known exposure to wildlife or farm animals, had never travelled abroad and had no relevant medical history; specifically, no reported previous bleeds (post‐spay or trauma) or exposure to toxins (chemotherapy, oestrogens, antibiotics, radiation, plants and snake bites).

The dog initially presented to the referring veterinarian for minor oral bleed of several days’ duration and presence of blood in her stools (nature not specified, digested or fresh). Physical examination confirmed gingival bleeding and revealed multiple petechiae (aural, oral and abdominal). Investigations conducted at the time documented a severe thrombocytopenia (total absence of platelets on the analyser, confirmed on an in‐house blood smear). Thoracic radiographs, abdominal ultrasound and urine sediment were reported unremarkable. Urine culture from a free‐catch sample was negative. Biochemistry revealed a non‐specific mild elevation in alanine aminotransferase (ALT 219 UI/L, reference interval: 10–125) and normal coagulation assays (prothrombin time 8.1 s, reference interval: 7.9–11.5; partial thromboplastin time 11.5 s, reference interval: 11–15). Treatment was initiated with immunosuppressive doses of prednisolone (50 mg/m^2^ of body surface area [body surface area in meter square = 10.1 × (weight in grams)^2/3^) ÷ 10,000], per os (PO), once daily (SID), Prednidale 5 mg, Dechra, UK) (Nam et al. 2017; Purcell et al. 2023). Within 24 h, observation of melena prompted the addition of sucralfate (1 g, PO, every eight hours (TID), Antepsin oral suspension 20%, Baldacci, Italy) and omeprazole (0.7 mg/kg, PO, twice daily (BID), Omeprazole 20 mg, Teva, UK). After 48 h, the dog presented again with ongoing melena, petechiae, pallor, lethargy and bounding pulses. Development of a marked, presumed pre‐regenerative, normocytic, normochromic anaemia on repeat haematology (haematocrit 11%, reference interval: 37–62), alongside haemodynamic instability and persistent complete thrombocytopenia (platelet 0 × 10^9^/L, reference interval: 148–484) precipitated referral.

Upon referral, physical examination confirmed the previous findings of pallor, multiple petechiae and low‐grade gingival bleed. Heart rate and respiratory rates were mildly elevated (120 and 40 bpm, respectively) with bounding pulses. A 2/6 left systolic murmur was audible on auscultation, and bronchovesicular sounds were normal. Rectal temperature was normal (38.3°C) with a mild amount of melena noted on the thermometer. Urine was grossly haematuric. The dog's overall clinical bleeding score (DOGiBAT) on admission was 8/18 (Makielski et al. 2018). The rest of the examination was unremarkable, including abdominal palpation, retinal assessment and blood pressure (127 mmHg average systolic, Doppler method).

Upon admission, point‐of‐care ultrasound ruled out cavitary effusion. Blood gas analysis was within normal limits, and manual packed cell volume (PCV) was 13%. Blood type was DEA 1 negative. Haematology confirmed the severe anaemia (haematocrit 12%, reference interval: 39–55), now markedly regenerative (reticulocytes 255 × 10^9^/L, reference interval: 0–60), revealed a mild neutrophilia with left shift and mild toxic changes (non‐segmented neutrophils 1.6 × 10^9^/L, reference interval: 0–0.1) and severe thrombocytopenia (5 × 10^9^/L, reference interval: 200–500), which was confirmed on blood smear. In‐house in saline agglutination test, Coomb's test, bedside test for angiostrongylosis (Angio Detect, IDEXX, Westbrook, USA) and vector‐borne diseases (SNAP 4DX, IDEXX, Westbrook, USA) were negative. Repeat imaging performed by a resident working under the supervision of a board‐certified diagnostic imaging specialist failed to unveil an underlying trigger (thoracic radiographs, abdominal ultrasound). Urine obtained by free catch was inadequately concentrated (specific gravity of 1.017), consistent with recent administration of prednisolone, and positive for blood on dipstick. Sediment was inactive but showed numerous well‐preserved erythrocytes.

The mildly elevated heart rate and tachypnoea were presumed secondary to the decreased oxygen‐carrying capacity caused by the marked anaemia and suggested transfusion dependency. This hypothesis was further supported by the bounding pulses, although lactates remained within normal limits. Other differentials for borderline tachycardia and mild tachypnoea included stress or pain. Congestive heart failure, hyperthermia, hypotension or severe lung disease had been ruled out during admission or by previous investigations.

The magnitude of thrombocytopenia was suggestive of immune‐mediated destruction (Brooks et al. 2024). Breed‐associated thrombocytopenia was not relevant (not reported in Dobermans). History ruled out envenomation and gestational thrombocytopenia. Parasitic causes (angiostrongylosis, dirofilariasis) and tick‐borne pathogens (anaplasmosis, ehrlichiosis, borreliosis), although deemed unlikely based on the absence of reported tick bites and travel outside of the United Kingdom, were ruled out by bedside antibodies and antigen assays. Splenic sequestration, losses through haemorrhage, intoxication (rodenticides), disseminated intravascular coagulation, haemophagocytic syndrome and many associative causes of immune‐mediated thrombocytopenia (infectious focus, neoplasia, drug‐associated) were rendered less likely by investigations conducted by the referring veterinarian. The heart murmur was presumed secondary to the marked anaemia, and the presence of microangiopathy (endocarditis) was not specifically investigated. Finally, bone marrow disorders (aplasia, phthisis, dysplasia, maturation arrest) were deemed unlikely based on normal cell counts of other lineages. Therefore, a diagnosis of suspected non‐associative immune thrombocytopenia (ITP) was made.

The initially non‐regenerative, normocytic normochromic anaemia was presumed pre‐regenerative and secondary to gastrointestinal bleed (melena). This was supported by the marked regenerative response observed on repeat haematology four days later. Other less likely differential diagnoses for markedly regenerative anaemia included haemolysis from immune‐mediated destruction (associative or not, e.g., Evan's syndrome) or oxidative damage. The absence of biochemistry or urinary signs of haemolysis, ghost cells or spherocytes on pathologist blood smear assessment and negative in saline and Coomb's tests made immune‐mediated haemolytic anaemia very unlikely. Similarly, the absence of Heinz bodies on the blood smear was not supportive of oxidative damage.

The dog was diagnosed with ITP and immediately received a transfusion of packed red blood cells (392 mL of DEA 1 negative blood, 14 mL/kg, PCV post‐transfusion 22%), a single injection of vincristine (0.02 mg/kg IV), alongside anti‐nausea (maropitant 1 mg/kg IV SID, Prevomax, Dechra, UK) and immunosuppressive doses of corticosteroids (dexamethasone 0.3 mg/kg IV SID initially, Colvasone 0.2% w/v, Norbrook, UK, followed by prednisolone 50 mg/m^2^ PO, SID). After 72 h of hospitalization, platelet count was persistently low (2 platelets per smear), the clinical bleeding score (DOGiBAT) remained unchanged (8/18) and the dog was quieter with hyperkinetic pulses. Haematocrit had decreased to 16%. A second blood transfusion was administered (DEA 1 negative packed red blood cells 314 mL, 11 mL/kg, PCV post transfusion 26%) and a second‐line immunosuppressant (mycophenolate mofetil 250 mg capsules, Tillomed, UK; 10 mg/kg PO BID) was implemented. Within 24 h platelet count started increasing (3 platelets per high power field), and so did haematocrit (30%). Upon discharge (Day 6), treatment was unchanged (oral prednisolone and mycophenolate), platelets were normal, and the heart murmur was no longer audible (Table 1). Owners were advised not to feed any raw treats for the duration of immunosuppressive treatment.

**TABLE 1:** Selected serial clinical, haematology and biochemistry parameters in a 5‐year‐old female neutered Dobermann diagnosed with presumed reactivation of *Neospora caninum* following immunosuppression for the management of immune thrombocytopenia.

	Prior to referral 24/11/23	Admission 27/11/23	Discharge 02/12/23	Recheck 1 12/12/23	Recheck 2 21/12/23	Recheck 3 12/01/24	Recheck 4 02/02/24	Recheck 5 01/03/24	Recheck 6 26/06/24
Body weight (kg)	28.4	27.95	25.2	24	24.2	26	25.3	25	—
BCS	—	5/9	4/9	3/9	4/9	5/9	5/9	5/9	—
Muscle condition^c^	—	1/4	—	3/4	2/4	1/4 (except face)	1/4 (except face)	1/4 (except face)	—
*Neospora caninum* (IFA)	—	—	—	> 1600	—	> 1600	> 1600	—	1:400
*Toxoplasma gondii* (IgG, IgM)	—	—	—	1:100, 1:80	—	—	—	—	—
Haematocrit (%)	39	12	30	37	32	41	49	42	44
Manual platelet count (× 10^9^/L)	0	0	200	> 500	> 500	250	350	200	247
Globulins (g/L)	38	—	—	28.6	43.3	43	36.5	—	27.3
ALT (U/L)	219	—	—	1585	628	62	42	—	55
AST (U/L)	—	—	—	1012	484	56	—	52^a^	—
CK (U/L)	—	—	—	7715	3424	241	105	415^a^	172
CRP (mg/L)	—	—	—	67.9	21.8	16.1	—	—	< 5
Prednisolone (mg/m^2^/day)	50	50	50	6	Stopped	—	—	—	—
Mycophenolate (mg/kg/day)	—	—	20	20	20	10	5^b^	Stopped	—
Clindamycin (mg/kg BID)	—	—	—	20	20	20	20	Stopped	—

^a^ Different analyser.

^b^ dose was decreased to 10 mg/kg every other day.

^c^ According to WSAVA—Muscle condition score chart.

Upon re‐assessment, 10 days later, the dog was reported lethargic, reluctant to exercise, with a swaying gait, severe muscle wastage and marked polyuria and polydipsia. Physical examination confirmed the generalized muscle wastage and documented weight loss (−1.2 kg, equivalent to 5% body weight) despite normal appetite. Neurological examination showed normal mentation, wide based stance, and stiffness but no neurological deficits, pain or joint effusion. Neurolocalisation was suggestive of peripheral myopathy/neuropathy. Retinal examination was normal. Differential diagnoses considered at this point included iatrogenic causes (prednisolone catabolism, acute steroid myopathy (Haran et al. 2018), mycophenolate myopathy (Galindo et al. 2005)) or exacerbation of a previously undetected condition including infectious (urinary tract infection, pyelonephritis, discospondylitis, endocarditis or protozoal myositis) and non‐infectious causes (disk disease and wobbler's syndrome). Repeat haematology showed appropriate platelet count (695 × 10^9^/L, reference interval: 200–500) and mature neutrophilia (neutrophils 23 × 10^9^/L, reference interval: 3.6–12). Biochemistry revealed markedly raised ALT (1585 U/L, reference interval: 21–102) and creatinine kinase (CK, 7715 U/L, reference interval: 50–200) alongside moderately increased C‐reactive protein (67.5 mg/L, reference interval: 0–5) and only marginally increased alkaline phosphatase (ALP 137 U/L, reference interval: 20–60) (Table 1). Bilirubin was within normal limits (2.4 umol/L, reference interval: 0–6.8). Measurement of serum aspartate aminotransferase (AST) was requested to help discriminated between hepatic or muscular elevation in ALT. Increased AST (1012 U/L reference interval: 0–50) was suggestive of the latter. Urine sediment was inactive. Serology for *T. gondii* was equivocal (IgG 1:100, reference <1:50 and IgM 1:80, reference < 1:20, clinical toxoplasmosis is typically associated with IgM > 1:256 and/or IgG > 1:512 (Lappin et al. 1989)) but showed high antibody titres (IgG) against *N*. caninum (indirect fluorescent antibodies > 1600). The dog was presumptively diagnosed with neosporosis and treatment with clindamycin (20 mg/kg PO BID, Zodon 264 mg, Ceva, France) was immediately initiated. The muscle wastage was imputed to a combination of corticosteroid side‐effects and neuromuscular neosporosis. To balance the need for immune suppression but try to limit wider *N. caninum* dissemination prednisolone was rapidly tapered (physiological doses, 0.2 mg/kg PO SID, for 7 days, before complete discontinuation) while mycophenolate (10 mg/kg PO BID) remained unchanged. Repeat examination at 2‐weeks, 5‐weeks, 2‐months, 3‐months and 4‐months showed a gradual resolution of the muscle wastage (except for the temporal muscle atrophy), weight gain, improvement in demeanour, energy levels and gait. *N. caninum* antibody titres were repeated after one and two months of treatment and remained markedly increased (> 1600, limit of detection of the laboratory). Gradual tapering of mycophenolate was achieved over 3 months. Clindamycin was discontinued after 12 weeks. At the time of writing (6 months after diagnosis of neosporosis) the dog has not experienced any long‐term consequences of *N. caninum* infection (no appendicular muscle atrophy), *N. caninum* titres have decreased (1/400) and the ITP remains in remission.

## 3 Discussion

Reactivation of opportunistic parasites like *T. gondii* and *N. caninum* is a well‐known complication of immunosuppressive treatments in small animals (Donahoe et al. 2015; Dubey et al. 2020). In the present case, reactivation of the parasite is suspected based on the historic consumption of raw food products and the very unlikely risk of acute infection in a dog fed strictly a dry commercial diet but reactivation of chronic asymptomatic neosporosis resulting from vertical transmission is also possible.

Neosporosis is often asymptomatic in adult dogs whereas vertical transmission or post‐natal exposure of puppies can result in clinical signs (polyneuritis, forelimb muscular atrophy and rigidity) (Silva and Machado 2016; Barber and Trees 1998). Seroconversion occurs within 2–3 weeks of exposure, but duration of immunity is unknown (Sykes et al. 2021). In naturally infected puppies, antibodies were documented as early as 5‐weeks of age and persisted for up to 5 years (Barber and Trees 1998). In the present case, the absence of gait anomaly in the dog's history makes congenital neosporosis less likely but not impossible as a significant proportion of puppies born from infected dam become serologically positive but do not develop clinical neosporosis (Barber and Trees 1998). In keeping with the low risk postulated in the immune mediated haemolytic anaemia (IMHA) and ITP consensus statements, *T. gondii* and *N. caninum* titres are not part of the standard investigations of ITP in our institution, unless prompted by suggestive clinicopathological changes (signs of polymyositis, polyneuropathy, elevated CK) and were therefore not run upon initial presentation (Garden et al. 2019; LeVine et al. 2024). Documentation of low serum titres prior to immunosuppression and fourfold increase on paired serology 2‐weeks later would have further supported the suspicion of protozoal reactivation (Silva and Machado 2016). Nevertheless, despite positive titres persisting for years in asymptomatic exposed dogs, the presence of titres superior or equal to 1:800 is rare in healthy dogs and deemed highly suggestive of clinical neosporosis when accompanied by adequate clinical signs (Barber and Trees 1996).

Contrarily to *T. gondii*, for which both IgG and IgM titres are measured allowing educated guesses on the animal's status (exposure vs. active infection), most laboratories only return IgG *N. caninum* titres. With *N. caninum* (IgG) serological titres ranging from 1:50 to 1:3200 in asymptomatic naturally infected dogs, discrimination between clinical disease and past exposure is difficult (Barker et al. 2021). Thus, clinical context is essential and pre‐existing seral reactivity is unlikely to preclude immunosuppression. However, if positive serological status is known, these cases should be monitored cautiously for protozoal reactivation.

Reactivation of *T. gondii* has been regularly documented in cats undergoing immunosuppressive therapy or immunocompromised (Ludwig et al. 2021; Salant et al. 2021; Moore et al. 2022; Fietz et al. 2023; Lo Piccolo et al. 2019, de Jesus Pena et al. 2017; Evans et al. 2017; Last et al. 2004; Beatty and Barrs 2003; Bernsteen et al. 1999) but experimental studies failed to confirm these observations (Lappin et al. 2015). Infection of felines by *T. gondii* prior to immunosuppression with ciclosporin did not result in reactivation whereas experimental infection during treatment caused clinical toxoplasmosis (Lappin et al. 2015). In response to such evidence, testing prior to immunosuppression in cats is not essential but exposure to toxoplasmosis should be avoided during treatment (ideally kept indoor, no raw meat) (Colombo and Sartori 2018). In the present case, the owners were advised to strictly avoid raw feeding and closely monitor the dog. While we cannot formally rule out that clinical neosporosis resulted from exposure during immunosuppression, it is considered highly unlikely.

Reactivation of *N. caninum* following immunosuppression has also been documented in the literature (14 cases). Diagnosis relied on direct visualisation of protozoal cysts on post‐mortem (*n* = 5), on skin cytology or histopathology (*n* = 7), or on liver fine needle aspirates (*n* = 1) followed by immunohistochemistry or polymerase chain reaction (PCR) to discriminate between *Sarcocystis* spp, *T. gondii* and *N. caninum* (La Perle et al. 2001 May; Hoon‐Hanks et al. 2013; Magaña et al. 2015; Curtis et al. 2020; Newton and Manens 2018; Legnani et al. 2016; Galgut et al. 2010; Ordeix et al. 2002; Fry et al. 2009; Mann et al. 2016; Gupta et al. 2011, Schuyter et al. 2013). In the last case, muscle biopsies revealed the presence of protozoan cysts and antibody titres for *T. gondi* (IgM 1:20, IgG 1:200) and *N. caninum* (> 1:800) were deemed most compatible with neosporosis (Serrano and Freeman 2017). When reported, antibody titres in dogs diagnosed with neosporosis secondary to reactivation following immunosuppression ranged from 1:80 to 1:6400. In the present case, diagnosis relied strictly on supportive clinicopathological changes (muscle wastage, gait anomaly, elevated serum ALT, AST and CK) alongside persistently elevated indirect fluorescent antibody (IFA) titres. This assumption is supported by recent literature on neuromuscular neosporosis documenting that although a multimodal diagnostic approach should be preferred, serology outperformed other modalities (molecular identification or histopathology with subsequent immunohistochemistry or in‐situ hybridation) in identifying positive cases (Alf et al. 2024). Similarly, a marked elevation of AST and CK is used as a screening tool to increase clinical suspicion of neosporosis in cases on meningoencephalitis of unknown origin (Jones and Harcourt‐Brown 2022). With a neurological examination supportive of peripheral neuromuscular disease, biopsy of muscle and PCR for *N. caninum* could have provided direct and/or molecular confirmation of the diagnosis (Fisher et al. 2024). This was carefully considered but given the constellation of findings, recent immunosuppression, history of raw feeding and ultimately elevated IFA titres, we elected not to pursue muscle biopsy on this balance of probability, risks (poor healing due to corticosteroids, opportunistic infection at the surgical site) and the potential for false negative due to the sporadic distribution of protozoal cysts across tissues (Fisher et al. 2024; Barber et al. 1996). Similarly, the pattern of increase of ALT and AST alongside the steep increase of CK made muscle damage more likely but, in absence of cytology or histopathology, hepatocellular damage secondary to protozoal hepatitis cannot be fully excluded. Fine‐needle aspirates of the liver are reported to have a low complication rate but were not pursued for similar reason as muscle biopsies (Cray et al. 2023). Nevertheless, the minimal evidence of cholestasis (ALP marginally increased, bilirubin within normal limits) contrarily to previous reports made protozoal hepatitis less likely (Hoon‐Hanks et al. 2013; Fry et al. 2009).

Discrimination between exposure and acute infection relies on supportive clinicopathological signs, including measurement of paired antibody titres, with an expected 4‐fold increase over 15–30 days (Silva and Machado 2016). In the present case, *N. caninum* titres were repeatedly high (highest laboratory dilution), which precluded formal confirmation of the acute status of the infection but were highly suggestive. As previously stated, the kinetic of antibodies and duration of immunity in cases of neosporosis is unknown, as are the effects of immunosuppressive therapy on their development. None of the previously described cases of neosporosis reactivation had repeated titres. Thus, it appears that assessment of the response to treatment cannot be based on follow‐up serology. In the present case, appropriate response to therapy was based on improvement of clinical signs and clinicopathological data (ALT, AST, CK, CRP) (Table 1). However, repeat measurement of *N. caninum* antibodies 3 months after discontinuation of medical management documented a marked reduction in titres.

The dog demonstrated a mildly increased *T. gondii* IgM and IgG. While this serological pattern could support an acute infection, clinical toxoplasmosis is commonly associated with higher titres (IgM > 1:256 and IgG > 1:512) (Lappin et al. 1989). In the present case, we suspect that the *T. gondii* titres were a false positive due to cross‐reactivity between assays for *T. gondii* and *N. caninum* (Gondim et al. 2017). PCR could have helped discriminate between aetiological agents but was not performed in this patient. Although it is regrettable that *T. gondii* titres were not repeated, with similar treatment modalities (clindamycin, TMPS, pyrimethamine) for both protozoal infections, this was less clinically relevant and testing would have been academic (Sykes et al. 2021).

The treatment of neosporosis has been based on the historic successful treatment of *T. gondii* (Barber and Trees 1996). Clindamycin has shown efficacy against *N. caninum* both in vitro and in vivo (Barber and Trees 1996; Lindsay et al. 1994). It is effective in suppressing the replication and dissemination of tachyzoites but does not appear to be effective against encysted bradyzoites (Dubey et al. 2007). Prolonged courses are required to improve clinical signs, with a minimum of 4 weeks recommended (10–12 mg/kg PO, every 8 h), but greater duration (more than 8 weeks) are often needed (Silva and Machado 2016; Sykes et al. 2021). Despite prolonged treatment, the risk of relapse remains (Fisher et al. 2024). Other reported drug combinations include TMPS as monotherapy (15 mg/kg PO, every 12 h), clindamycin with TMPS, TMPS with pyrimethamine (1 mg/kg PO, every 24 h), clindamycin with pyrimethamine and rarely toltrazuril for refractory cases (Silva and Machado 2016; Sykes et al. 2021; Barber and Trees 1996; Strohbusch et al. 2009). In cases with central nervous system involvement, TMPS should be considered due to its good penetration into the central nervous system, although there is little evidence to suggest that it would improve outcomes (Ineichen et al. 2020). In the present case, treatment with TMPS was not considered based on the risk of hypersensitivity in Doberman Pinschers, which can lead to the development of non‐septic polyarthritis, glomerulonephropathy and haematological dyscrasias (Giger et al. 1985). Other potential adverse effects of sulphonamides not exclusive to Doberman Pinschers include fever, skin eruptions, hepatopathy, keratoconjunctivitis sicca and hypothyroidism (Anyogu et al. 2017; Trepanier 2004). These reactions can occur both idiosyncratically and with prolonged treatment.

Among the 14 previously reported cases of *N. caninum* reactivation, only six dogs made a recovery; four diagnosed with strictly cutaneous neosporosis (e.g., with skin lesions but no other signs or organ affected) (Morganti et al. 2024; Newton and Manens 2018; Galgut et al. 2010; Gupta et al. 2011), one with protozoal hepatitis (Curtis et al. 2020) and one with neuromuscular disease (Fry et al. 2009). The four cases of cutaneous neosporosis were all treated strictly with clindamycin (11–20 mg/kg PO BID), for several weeks, alongside prednisolone (anti‐inflammatory doses) (Morganti et al. 2024), ciclosporin (5 mg/kg PO SID) (Newton and Manens 2018) or discontinuation of immunosuppressive drugs (Galgut et al. 2010; Gupta et al. 2011). The two cases of systemic neosporosis (protozoal hepatitis and neuromuscular disease) were successfully managed by discontinuation of immunosuppression and administration of TMPS and pyrimethamine in the first case (Curtis et al. 2020) or in addition of clindamycin in the second (Fry et al. 2009). Out of the eight reactivation cases that died, five were diagnosed post‐mortem, precluding any retrospective reflection on their potential response to treatment with or without withdrawal of immunosuppression (La Perle et al. 2001 May; Hoon‐Hanks et al. 2013; Dubey et al. 2020; Legnani et al. 2016; Ordeix et al. 2002). One was euthanized before treatment was implemented (Mann et al. 2016). The last dog was diagnosed with cutaneous neosporosis in a context of hyperadrenocorticism. Treatment with low dose of clindamycin (6 mg/kg PO BID) allowed a temporary resolution of the skin lesions before recurrence, 40 days later when the dog was euthanized for possibly unrelated reasons (development of anorexia and diarrhoea) (Serrano and Freeman 2017). Thus, early recognition of the disease and implementation of treatment without delay appears critical for the successful management of *N. caninum* reactivation. In the present case, the presumed neuromuscular neosporosis was successfully managed using solely clindamycin despite ongoing immunosuppression with mycophenolate. The limited number of reports describing the outcome of dogs diagnosed with neosporosis reactivation following immunosuppression precludes any comment on the requirement for discontinuation of immunosuppression. In absence of consensus, rapid tapering of immunosuppression should be considered while balancing the risk of relapse of concurrent immune‐mediated comorbidities.

In immunocompetent dogs, successful management (complete recovery) of neosporosis has only been reported in 12 adult dogs, one presenting with presumed protozoal myocarditis and 11 with neuromuscular signs (polymyositis *n* = 4, neurological signs *n* = 7) (Alf et al. 2024; Fisher et al. 2024; Thate and Laanen 1998; Didiano et al. 2020; Agudelo et al. 2016). Most were treated with a combination of clindamycin (12.5 mg/kg PO BID) and trimethoprim/sulfamethoxazole (15 mg/kg PO BID) for up to 6 months (*n* = 5) (Didiano et al. 2020). However, five cases made a complete recovery with clindamycin alone, the adult dog diagnosed with protozoal myocarditis (Agudelo et al. 2016) and four with neuromuscular disease (Alf et al. 2024; Fisher et al. 2024). In the latter, one dog also received corticosteroids (dose not stated) (Fisher et al. 2024). A single case of congenital neosporosis, presenting with typical hind limb muscular atrophy and stiffness, describes almost complete recuperation (gait 90% normal) following treatment with clindamycin and physiotherapy for 18 months (Crookshanks et al. 2007). Together, these cases and ours, suggest that in specific neosporosis cases (cutaneous, neuromuscular, myocardial, early‐stage paediatric onset) clindamycin could be sufficient as sole therapy.

Unlike previous reports that describe significant morbidity and mortality in immunosuppressed protozoal reactivation, the dog described in this report responded well despite ongoing immune suppression (La Perle et al. 2001 May; Hoon‐Hanks et al. 2013; Dubey et al. 2020; Legnani et al. 2016; Ordeix et al. 2002; LeVine et al. 2024; Barber and Trees 1996; Salant et al. 2021). The possibility of acute steroid myopathy was considered as an alternative explanation. Slow gradual muscle atrophy secondary to endogenous excess of glucocorticoids is commonly described in veterinary medicine whereas muscle loss following exogenous glucocorticoid administration has only recently been investigated and quantified (Trepanier 2004; Thate and Laanen 1998; Didiano et al. 2020). Chronic steroid‐induced iatrogenic myopathy is also well recognized in human medicine (Purcell et al. 2023). In human medicine a rare, acute form of glucocorticoid induced myopathy has been described (less than 20 case reports). This acute myopathy is defined as an acute‐onset of muscle weakness but only applied when no alternative explanation exists in patients started on glucocorticoid treatment in the preceding 2 weeks (Purcell et al. 2023). The clinical presentation ranges from mild proximal limb weakness to ventilation dependency (Purcell et al. 2023; Agudelo et al. 2016). Of note, muscle enzymology is often unremarkable in human acute glucocorticoid myopathy. Likewise, muscle biopsy and electromyography abnormalities are nonspecific (Purcell et al. 2023; Agudelo et al. 2016). Outcomes are variable, complete recovery can occur within 3–4 weeks of tapering steroids but may also take several months and some individuals experience irreversible muscle damage. This variable presentation, progression and nonspecific diagnostic test results make acute steroid myopathy a challenging disorder to diagnose (Purcell et al. 2023). Acute steroid myopathy has to the authors’ knowledge never been described in dogs. While acute steroid myopathy could account for the muscle wastage, the ataxia and the marked elevation in markers of muscle damage are not typical (Purcell et al. 2023; Agudelo et al. 2016). Moreso, the very high *N. caninum* titres and serological response to clindamycin treatment support protozoal disease. Since an acute infection with *N. caninum* is highly unlikely in a dog strictly fed a commercial dry diet, the authors favoured the hypothesis of protozoal reactivation (from past raw meat consumption or vertical transmission).

In conclusion, this case report describes the successful treatment of presumed *N. caninum* reactivation, with clindamycin, in a dog that was concurrently immunosuppressed for ITP. Although acute steroid myopathy cannot be completely ruled out, this report suggests that early recognition of protozoal reactivation and aggressive therapeutic management (diminution of immunosuppression, antibiotic therapy and cautious monitoring) could result in positive outcome.

## Author Contributions

**Perrine Henry**: writing – original draft, conceptualisation, methodology, data curation, investigation. **Paul Rees**: writing – review and editing, data curation. **Tyler Morrison**: investigation, writing – review and editing. **Glynn Woods**: conceptualisation, investigation, validation, supervision.

## Ethics Statement

The authors have nothing to report.

## Consent

Consent for publication was granted as part of our hospital standard admission process.

## Conflicts of Interest

The authors declare no conflicts of interest.

### Peer Review

The peer review history for this article is available at https://publons.com/publon/10.1002/vms3.70425

## Data Availability

Additional information available upon request.
